# Cluster Analysis of Dry Eye Disease Models Based on Immune Cell Parameters – New Insight Into Therapeutic Perspective

**DOI:** 10.3389/fimmu.2020.01930

**Published:** 2020-09-29

**Authors:** Chit Tong Lio, Sandeep Kumar Dhanda, Tanima Bose

**Affiliations:** ^1^Chair of Experimental Bioinformatics, Technical University of Munich, Munich, Germany; ^2^La Jolla Institute for Immunology, La Jolla, CA, United States; ^3^Institute for Clinical Neuroimmunology, Ludwig Maximilian University of Munich, Munich, Germany

**Keywords:** dry eye disease, animal models, helper T cells, inflammation, therapy

## Abstract

Dry eye disease (DED) can be represented as a display of disease in the mucosal part of the eye. It is quite distinct from the retinal side of the eye which connects with the neurons and thus represents the neuroimmunological disease. DED can occur either by the internal damage of the T cells inside the body or by microbial infections. Here we summarize the most common animal model systems used for DED relating to immune factors. We aimed to identify the most important immune cell/cytokine among the animal models of the disease. We also show the essential immune factors which are being tested for DED treatment. In our results, both the mechanism and the treatment of its animal models indicate the involvement of Th1 cells and the pro-inflammatory cytokine (IL-1β and TNF-α) related to the Th1-cells. The study is intended to increase the knowledge of the animal models in the field of the ocular surface along with the opening of a dimension of thoughts while designing a new animal model or treatment paradigm for ocular surface inflammatory disorders.

## Introduction

Dry eye disease (DED) is characterized by the inflammation of the ocular surface and it involves the structures present in the mucosal portion of the eye like conjunctiva, cornea, meibomian glands, goblet cells, and lacrimal glands. In humans, the disease is associated with painful itchy eyes followed by chronic progressive phenotype leading to reduced vision and blindness. The history of DED can be traced back to the early 1900s when a Danish physician depicted the importance of two subclasses; the primary Sjögren’s syndrome (pSS, related to dry eye or mouth) or the secondary Sjögren’s syndrome (sSS, related to autoimmune diseases like rheumatoid arthritis) (1). DED being a multifactorial disease in the tear film and ocular surface suggests a dysregulation of the immune mechanism and leads to a cycle of continued inflammation in its chronic form (2). Though the disease is quite clearly phenotyped in human subjects, the exact pathogenesis and underlying immune mechanisms have only recently been understood with the help of DED animal models. The animal models used for DED research are rodent (mice, rats), canine (dogs), porcine (pigs), feline (cats), other mammals (sheep, rabbits), and also non-human primate models (3, 4).

The choice of animal model is very much dependent on the question researchers are investigating. For example, mice are mostly used for genetic manipulation, rabbits for the pharmacological experiments and toxicological testing, feline, canine, and porcine models are used for the determination of pathological features similar to humans, like the blinking rate in Schirmer’s test. One of the most commonly used mice models is the NOD (non-obese diabetic) mice.

The environment plays a crucial role in DED because the ambient dehydrating environment can lead to changes in the disease phenotype and progression. That is why there are several DED animal models generated by modulating the ambient environment using, for example, a controlled-environment chamber or desiccating stress or scopolamine-induced models. Oxidative damage is induced in these animals with a change in the ambient environment like many other ocular disorders (e.g., cataract, acute macular degeneration). Mechanistically, the reactive oxygen species in these environment-induced dry eye models involve NLRP3 inflammasome activation and increasing IL-1β secretion through the activation of Caspase-1 (5). We have used both genetically modified and environment-induced DED models for our study, which are enlisted in the section “Materials and Methods.”

The involvement of both innate and adaptive immune system pathways in DED is well documented (6, 7). Several cytokine molecules are identified to regulate or to trigger these pathways. For example, topical treatment with IL-1R-antagonist in C57BL/6 mice ameliorates disease whereas inhibition of TNF-α in the salivary gland of NOD mice can have a negative effect on salivary gland function as shown in (8, 9). This makes this disease even more complicated to treat through a common pathway/molecule/immune factor.

We aim to find out the most commonly used animal models in DED followed by the search for the most important immune cell contributing to disease progression. In our result, a cluster analysis of different cell types, cytokine signatures and different animal models depict the importance of both CD4^+^ and CD8^+^ Th cells, Th1 cytokines IFN-γ and TNF-α and IFN-γ-related cytokine CXCL9. Along with the mechanism of DED, we have evaluated the interest of this research area after the analysis of research papers from all over the world. Finally, we showed the most important cytokine molecule (IL-6, IFN-γ, TNF-α, and IL-1β) used for the treatment of DED animal models. It is clear at least from the current study that the blocking of pro-inflammatory cytokine (IL-1β and TNF-α) and induction of anti-inflammatory cytokine (IFN-γ, IL-12, and IL-4) might help to ameliorate the disease. We also have observed a higher abundance of IL-6 in many of our animal models which might modulate both pro- and anti-inflammatory pathways. Thus, understanding of a delicate balance between Th1 and Th2 cells and their secreting cytokines will be enlightening for DED research.

Our purpose for the study is to find a common mechanism through which one can treat DED, one of the predominant diseases of the ocular surface. We hope that this analysis will open new possibilities of treatment in addition to the existing broad treatment options. Besides, this analysis will also help us to understand the interplay of different cytokines in DED and we might recommend a combinatorial treatment for DED. This can act as a cautionary measure for designing a drug based on a single cytokine and might prevent previous issues such as the exacerbation of diseases in multiple sclerosis patients with anti-TNF treatment (10). DED is also a complex disorder and scientists are still optimizing the diagnostic markers of DED and it is important to have a combinational approach when the disease etiology of DED is not clear.

## Materials and Methods

### Study Retrieval and Selection

**FIGURE 1:**
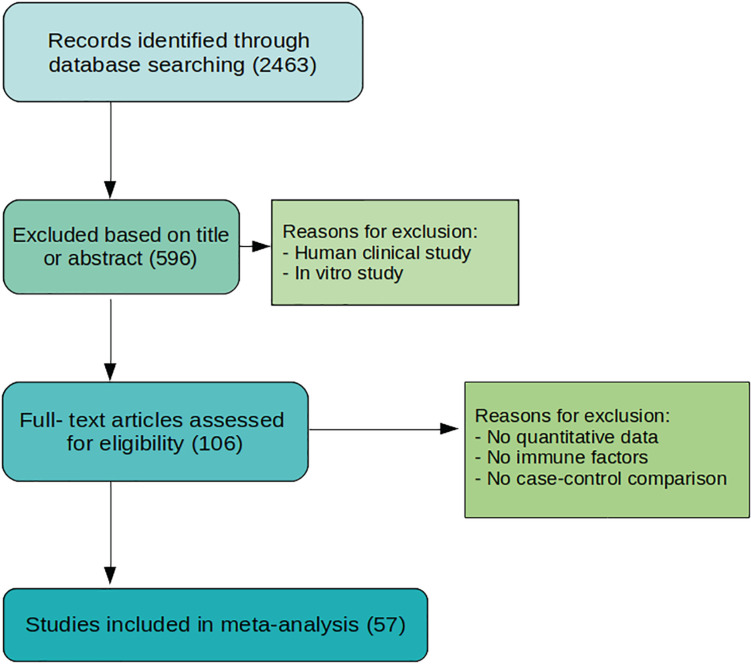
Flowchart of study selection process. The process originates with 2463 studies from searching through several databases. A total of 57 studies are selected for meta-analysis after evaluation and exclusion.

The process for study selection is summarized in Figure 1. Databases like Medline, PubMed, Embase, Google Scholar, and Web of Science were searched systematically. Our final analysis is based on 53 studies filtered using well-defined parameters at each step. DED was searched with keywords for animal models – mice, rabbit, canine (dogs), porcine (pigs), rats, sheep, feline (cats), horse, guinea pigs, monkeys (non-human primates), knock-in, knock-out (KO) animals; immune cells, cytokines and chemokines – IL-6, TNF-α, IL-1β, IL-21, IL-17, CCR1, CCR2, CXCR3, CXCR2, IFN-γ, IL-23, CD4^+^ T cells, CD8^+^ T cells, Th17, Th1, Th2 cells, B cells, monocytes, macrophages, B-regs, IL-10, T-regs, HLA, TLR, IL-2, IL-4, Tc, IL-17, CCR6, CD45RO, CD45RA, CCR7, CCL20, CD11c, CD14, CD16, CD19, CD3, CD20, BAFF, APRIL, TACI, MyD88, IL-1, TNF, IFN, CCR5, CCL, MCP, MIP, CCR2, CX3CR1, IL-12, natural killer cells, IL-13, FoxP3, CD25, TGFβ, TGF, IL-14, IL-5, dendritic cell, complement system, neutrophils, memory cells, γδ-T cells, innate lymphoid cells, plasma cells, intra-epithelial CD8^+^ lymphocytes, mucosal-associated invariant T cells, immunoglobulins (Igs), CD69, CD62L, CD103, M-CSF, GM-CSF, T-bet, GATA-3, CD44, CD45, CD27, CXCL10, CD40, CD28, IL-18, IL-1, TNF, IFN, and general terms interleukins, cytokines, antibodies, and lymphocytes as well. The exclusion process was first done by selecting articles according to title or abstract. Articles with patient participants and *in vitro* studies were excluded. Articles were then accessed fully, those without enough data, no immune factors included, or data that is not quantified, as well as no comparison to normal control, were excluded.

### Animal Models

C57BL/6 mice used in the studies are induced to dry eye by exposure in a controlled-environment chamber upon subcutaneous injection with scopolamine hydrobromide (8, 9, 11–24), 0.2% benzalkonium chloride (BAC) induction (25), and environmental desiccating stress (26–30). Mice models with C57BL/6 background are Thrombospondin 1 (TSP-1) conditional knockdown mice (31, 32), CD25 conditional knockdown (33–36), PD knock-in mice (37) and B6.NOD-Aec1Aec2 mice (38, 39). Another wild type mice model (DS mice) is used to generate a dry eye model by applying desiccating stress (40, 41).

The *Aire* deficient mice model has a phenotype similar to SS symptoms by knocking out the *Aire* transcription factor that is responsible for self-antigen expression regulation (42, 43).

Non-obese diabetic mice are immunodeficient mice that are prone to develop spontaneous autoimmune sialadenitis and exhibits SS (44–48). As an etiology, female mice develop autoimmune sialadenitis, whereas male mice develop dacryoadenitis and ocular surface inflammation. PSS is induced in NFS/N mice by performing thymectomy (49). CBA/J mice is a general-purpose model in which Botox-B (BTX-B) is injected to induce dry eye (50). In Albino Rabbit, 0.1% BAC eye drop is applied to induce dry eye (51–53). Another study used 1% atropine sulfate to instill into the eyes three times a day for 3 days (54). For Wister rats, Joossen et al., and Park et al., induced dry eye by removing the lacrimal gland (55, 56). Ru et al., induced dry eye by injecting scopolamine hydrobromide (57).

In Studies that used Lewis rats, Viau et al., and Han et al., induced the disease by injecting scopolamine hydrobromide (58, 59). Hou et al., induced the disease by injecting lacrimal gland extract from Sprague-Dawley rats to the Lewis rats (60). For Sprague-Dawley rats used in Hyun et al., the disease is induced by introducing Urban Particulate Matter (UPM) to the eyes (61).

In the analysis, these animal models are categorized into individual groups according to the animal strain. We have also performed the analysis categorizing the animal models according to the induction method to develop the DED (e.g., desiccating stress, Botox-B, benzalkonium chloride, UPM, and Atropine sulfate) but the overall result did not differ much (data not shown).

### Statistical Analysis

Data were analyzed using R package meta for meta-analysis. The script can be found on GitHub^1^. Github is an online repository used by bioinformaticians to store the data/code and this can be used by researchers in the future. Meta-analysis was performed for each of the animal models and the mean, standard deviation, and total number from the experimental and control group are analyzed from those animal models. To analyze all these data using meta-analysis models, a variance estimate telling how dispersed the data and effect size is required. The inverse variance (IV) method is the variance estimate that is taken inversely. IV weighting can resolve the inequality of the effect sizes among the studies by giving preferences to the larger effect size. To access the heterogeneity of the data, the *I*^2^ value is calculated denoting the percentage of variability of the pooled effect sizes within the analysis. The data with *I*^2^ values lower than 50% would be considered as coming from a homogeneous population, and the fixed-effects model would be used in this case; otherwise, the random-effects model was used. The fixed-effect model assumes the included studies have a higher variation, hence showing lower heterogeneity. These models resulted in a standard mean difference (SMD) with its 95% confidence interval (CI) for each group. Higher SMD indicates upregulation in the immune factors in animal models. Outliers with extreme effect sizes (>50 or <−50) are excluded to avoid distortion of effect estimates. The following Table 1 shows the excluded studies.

**TABLE 1:** Excluded immune factors and the corresponding studies.

**Study**	**Standard mean difference**
De Paiva 2006_TNFα	812.90323
De Paiva 2010_TGFβ	9654.30464
De Paiva 2010_CCL20	68.96188
De Paiva 2010_IFNγ	60.12935
Yoon 2007_CCR5	478.87324
Yoon 2007_CCR3	−107.81889
Huang 2018_IL23R	54.19355
Huang 2018_IL21	894.19355
Huang 2018_CCL20	3603.87097
Huang 2018_IFNγ	180.64516
Joossen 2016_IL2	234.83871
Joossen 2016_IL12	1345.80645

*Cases defined as outliers which the standard mean differences are higher than 50 or lower than −50.*

## Results

**FIGURE 2:**
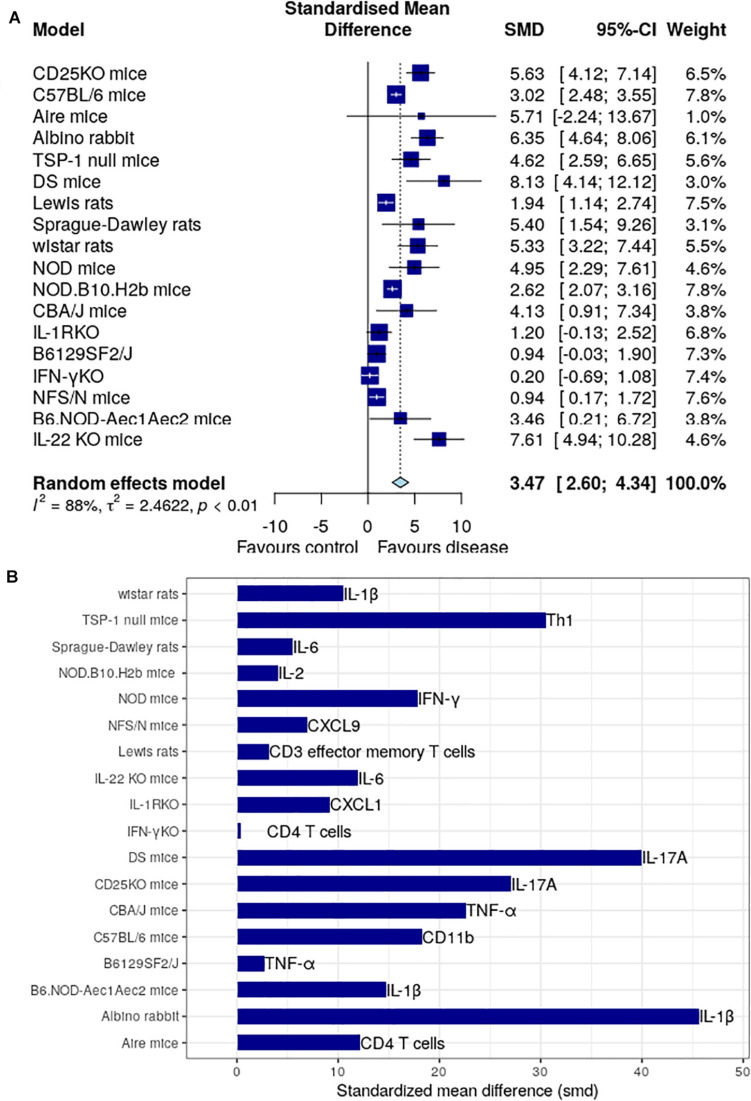
Meta-analysis of different immune factors in animal models for dry eye disease. **(A)** The global standardized mean difference for each of the immune factors is obtained by our proposed meta-analysis is represented by the blue square in the forest plot. The 95% confidence interval is shown by the interval line across the blue squares. DS mice has the highest SMD among all animal models, followed by CBA/J mice with TNF-α. Forest plots for each of the models are shown in the Supplementary Figures S1–S15. **(B)** The standardized mean difference of immune factor for each model that is most expressed. IL-17A is the highest in DS mice and TNF-α in CBA/J mice.

We have intensively studied 53 research papers, which were finalized for this specific study after a rigorous process of assessment shown in Figure 1 and described in the section “Materials and Methods.” Figure 2 determines the animal model, which is mostly used, and the immune factor, which is predominant in these chosen animal models. Figure 2A shows the use of CBA/J mice (SMD: 7.28) mostly in the studies related with inflammatory molecule followed by Albino rabbit (SMD: 6.35), CD25-KO mice (SMD: 5.63), Sprague-Dawley rats (SMD: 5.40), Wistar rats (SMD: 5.33), NOD mice (SMD: 4.95), TSP-1 null mice (SMD: 4.62), C57BL/d mice (SMD: 3.08), B6.NOD-Aec1Aec2 mice (SMD: 2.92), Lewis rats (SMD: 1.94), and least shown with B6129SF2/J and NFS/N mice (SMD: 0.94). Besides, the AIRE mice and DS mice show quite higher SMDs (5.71 and 8.13, respectively) but that is due to the overpowering effects of two papers (40, 43) as shown in Supplementary Figures 12, 15. Thus, we cannot consider these two animal models as the homogeneous distribution of the factors. The forest plots are described in detail in the Supplementary Figures 1–15. On the other hand, our observation shows a combination of pro-inflammatory and anti-inflammatory factors in those animal models. Among those molecules, IL-1β from Albino rabbit and Th1 type of immune cells from TSP-1 null mice are shown to be predominant (SMD: ∼45 and ∼30, respectively). This is followed by the presence of CD11b (SMD: ∼20) in environmental-factor-induced C57BL/6 mice models. In contrast, the environment-induced animal models (C57BL/6 an Albino rabbit) show the presence of innate immune cell features like dendritic cells (CD11b) and IL-6 (SMD: ∼20 for DED in Albino rabbit with BAC drops). Along with this, Th1-cell cytokine and chemokine IFN-γ, TNF-α, and CXCL9 seem to be quite predominant in the NOD (SMD: ∼20), CBA/J (SMD: ∼10), NFS/N (SMD: ∼5) mice, respectively. Besides, we could also find the presence of IL-1-cytokines in different other animal models (Wistar rats and B6.NOD-Aec1 Aec2 mice). As mentioned in the section “Materials and Methods,” we have though seen higher SMD in the case of IL-17A for the DS and CD25-KO mice (SMD: ∼40 and ∼30, respectively), but the distribution of this immune factor is not homogeneous. It is mostly the overpowering effects of the few papers (36, 40) as clearly shown in Supplementary Figures 3, 15.

**FIGURE 3:**
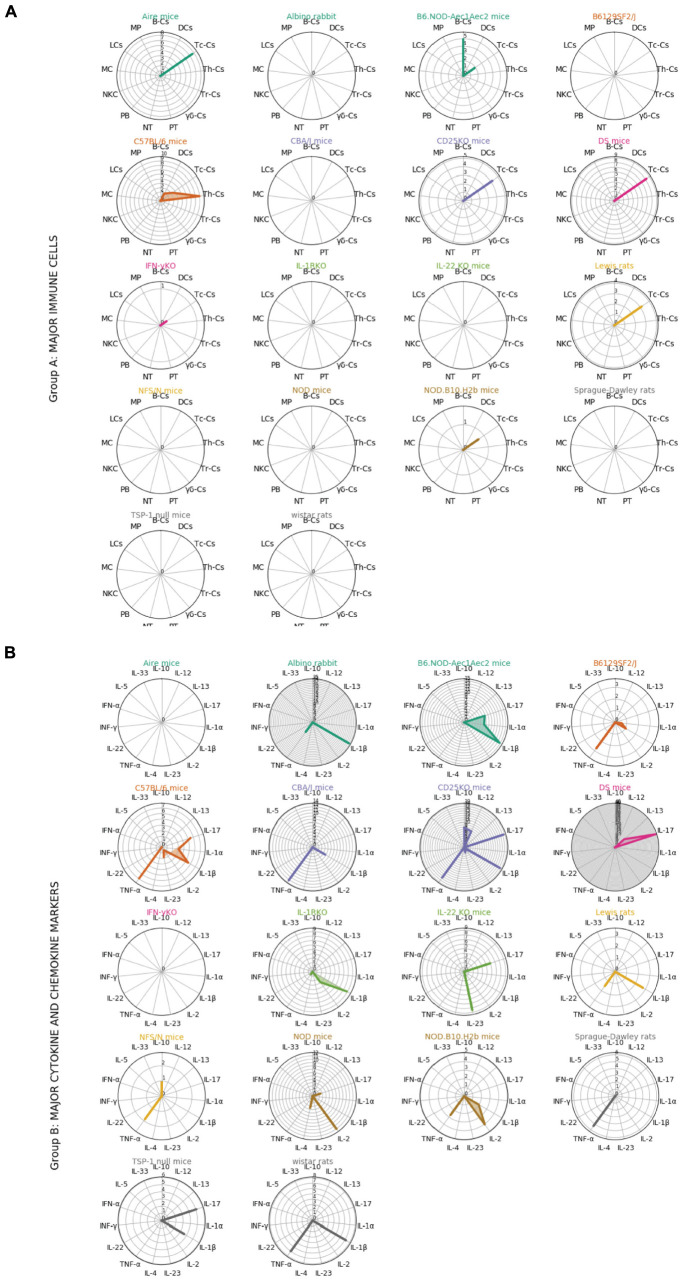
Radar plots showing the relationships between each animal models and groups of immune factors. Immune factors are grouped into 3 groups (Group A, major immune cells; Group B, major cytokine and chemokine markers; Group C, functions overlying on multiple cell types). Each color represents different animal model. The standardized mean difference between disease model and control from the meta-analysis for each immune factors in different animal models are plotted in radar plots (see Supplementary Material). Each circle in the radar plots represents an increasing of 1 in SMD. Th-Cs, T helper cells; Tc-Cs, T cytoxic cells; Tr-Cs, T resident cells; gb-Cs, gb cells; NT, neutrophils; PB, plasmablasts; NKC, natural killer cells; MC, mast cells; LCs, langerhans cells; MP, macrophages; B-Cs:, B cells; DCs, dendritic cells; PT, platelets.

In the radar plots of Figure 3, we followed a cluster analysis with three different groups of factors: group A with major immune cells, group B with major cytokine and chemokine markers, and group C with the functions overlaying on multiple cell types. We have only mentioned the significantly modulated factors in the section “Results” to avoid further complications. Some examples for group C are TLR4, IL-6, CD45, which are involved in both innate and adaptive immune system pathways. The radar plot in Figure 3 Group A depicts that CD4^+^ and CD8^+^ Th-cells play a predominant role in C57BL/6, B6.NOD-Aec1Aec2, Aire mice, DS mice, CD25-KO, and TSP-1 null mice. Apart from two Th cells, B cell is also shown to be important in this cluster represented by the TSP-1 null and B6.NOD-Aec1Aec2 mice. Some mice model radars in this cluster are empty reflecting the importance of specific analyzed populations in Figure 3 Group B. Here, we mostly observe the importance of Th1 cytokines IL-1β and TNFα represented by the Albino rabbit, Lewis rats, Sprague-Dawley rats, CD25-KO, B6 NOD-Aec1Aec2, B61295F2/J, C57BL/6, CBA/J, NFS/N, NOD, and TSP-1 null mice. The animal models influenced by the environmental factors can have the presence of both the cytokines related to the innate (IL-1β) and adaptive immune system (IFN-γ, TNF-α, IL-17, and IL-23) as depicted in the Albino rabbit and C57BL/6. Besides, the other T-cell types (Th17 and Tregs) shown to play a role in the B6.NOD-Aec1Aec2, C57BL/6, CD-25-KO, DS, NOD, NFS/N, TSP-1 null mice were represented by IL-17 and IL-10. In our calculation, we could mostly find the abundance of pro-inflammatory cytokines (IL-1β, TNF-α, and IL-2) with few animal models showing the anti-inflammatory cytokines (IL-10 and IL-4). Our finding of the predominance of Th1-related cytokines did not alter much in Figure 3 Group C. We could mostly find the importance of cytokine IL-6 in this cluster followed by TGF-β and CXCL9. We observe the presence of CD11b and TLR4 in C57BL/6 mice, which are an important component of the innate immune system.

**FIGURE 4:**
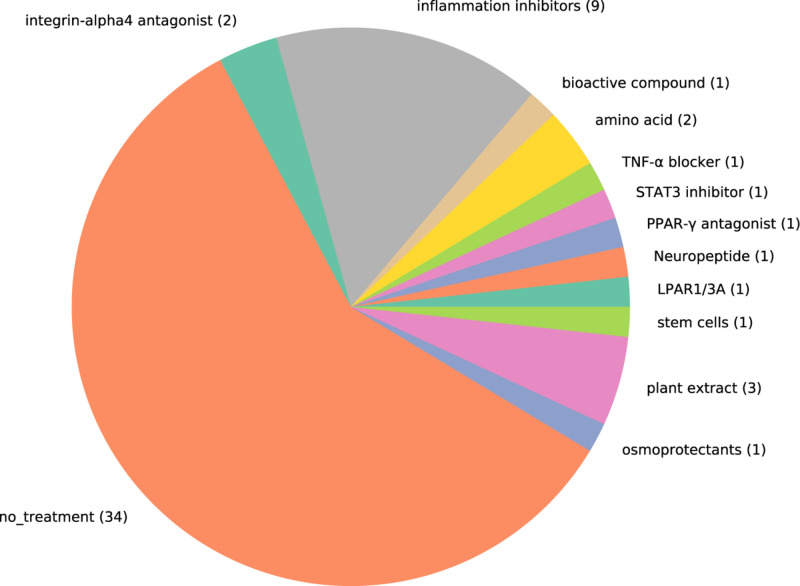
Pie chart showing the proportion of studies that include treatment for dry eye disease. Each treatment used in the studies are represented by one color. The number in the pie chart shows the number of studies that used the type of molecules to evaluate treating effect. MK2I, MK2 inhibitors; LPAR1/3A, LPAR1/3 antagonists.

To find the treatment molecule used in the selected papers, we showed Figure 4 and Table 2. As treatments related to the immune factors, we could see the use of anti-inflammatory drugs corticosteroids, doxycycline, TNF-α blocker HL036, and immunosuppressant MK2i (8, 10, 17). Other than the immune factors, the treatment paradigm includes osmoprotectants (betaine, L-carnitine, and erythritol), amino acid (cyclosporine A), integrin-α4 antagonist (dexamethasone), artificial tears (epigallocatechin gallate (EGCG), hyaluronic acid), plant antioxidant (10, 17, 26, 32, 51, 53). As it is shown from Figure 4 that the number of studies has not predominated in any case. It is a hint that a combinational treatment might be helpful in alleviating DED. Our effort here is not to find the list of treatments available in the literature but to show that there are still unspecific treatments (e.g., immunosuppressants, plant extract), which are recommended for this complex ocular surface disease. Thus, there is a huge gap in knowledge and treatment regimens for DED.

**TABLE 2:** Studies contain treatment effect.

**Study**	**Treatment**	**Type**	**Model**
(26)	Betaine, L-carnitine, erythritol	Osmoprotectants	C57BL/6 mice
(9)	Pioglitazone (PIO)	PPAR-γ antagonist	C57BL/6 mice
(11)	Mixed medicinal plant extracts	Plant extract	C57BL/6 mice
(51)	Cyclosporine A (CsA)	Amino acid	Albino rabbit
(31)	Novel antagonist GW559090	Integrin-α4 antagonist	TSP-1 null mice
(12)	Corticosteroids, doxycycline	Inflammation inhibitors	C57BL/6 mice
(52)	Epigallocatechin gallate (EGCG), hyaluronic acid	Inflammation inhibitors	Albino rabbit
(61)	Amygdalin	Bioactive compound	Sprague-Dawley rats
(61)	Apricot kernel extract	Plant extract	Sprague-Dawley rats
(15)	HL036	TNFα blocker	C57BL/6 mice
(55)	Cyclosporine A (CsA)	Amino acid	Wistar rats
(55)	Restasis and dexamethasone	Inflammation inhibitor	Wistar rats
(17)	Novel antagonist GW559090	Integrin-α4 antagonist	C57BL/6 mice
(44)	Topical TSG-6	Inflammation inhibitors	NOD.B10.H2b
(73)	Adiponectin	Inflammation inhibitors	C57BL/6 mice
(45)	Vasoactive intestinal peptide (VIP)	Neuropeptide	NOD mice
(50)	FK506	Inflammation inhibitors	CBA/J mice
(46)	Ki16425	LPAR1/3A	NOD mice
(56)	Polygonum cuspidatum (PCE)	Plant extract	Wistar rats
(25)	S31-201	STAT3 inhibitor	C57BL/6 mice
(57)	α-Melanocyte-stimulating hormone	Inflammation inhibitors	Wistar rats
(54)	CM-hUCESC	Stem cells	Albino rabbit
(53)	Epigallocatechin gallate (EGCG), hyaluronic acid	Inflammation inhibitors	Albino rabbit
(22)	MK2i	Inflammation inhibitors	C57BL/6 mice

*This table shows the molecules and the model used by studies that tested the effect of molecules as a mean of treatment.*

## Conclusion and Discussion

The functionality of CD4^+^ Th1 and Th2 subtypes are related to the presence of various cytokines. Detection of IL-2, IFN-γ, IL-4, and IL-5, associating with B cell accumulation, suggests a role of Th1 in disease induction and maintenance, and Th2 in disease progression. The Th1-associated pro-inflammatory cytokine IFN-γ is regulating the conjunctival apoptosis in desiccating stress models (62–65), and IL-7 upregulates the expression of IFN-γ (63). IL-13 as a Th2 cytokine is also proposed to be involved in disease pathology as shown in Id3^–/–^ mice (66). The role of Th17 cells was critically explored in SS in a chronic dry eye mouse model. Chronic ocular surface damage is mainly mediated by a memory T cell population, the response of which is predominantly mediated by Th17 cells (12). Co-transfer of CD8^+^CD103^+^ Treg had no effect indicating that CD8^+^ can suppress the initiation of pathogenic Th17 cells, but not the prolongation of disease (41). In our observation, we have found the involvement of the adaptive immune system pathways in all the animal models but the presence of innate immune system pathways only in the environment-induced animal models. This is a piece of important information while designing a drug for the treatment of DED. Current drugs for DED, which are in the Human study phase 2/3 clinical trials, are mostly related to the innate immune system pathways (67). This study enables us to identify the importance of the right environmental condition in which the molecules/cell types of adaptive immune system pathways are involved. Along with the essential balance between Th1 and Th2 cells with the pro- and anti-inflammatory cytokines, it is also interesting to note that IL-6 and TGF-β are promoting cytokines for Th17 cells, and therefore although by themselves are considered to be general, by blocking either or both of those, there will be considerable inhibiting effects downstream on the Th17 cells (68).

This study is also a trial to increase the awareness of the researchers working in this field. Awareness is twofold here: at first, there is no single or combination of studies in DED that depicts the exact picture of the modulation of several cytokines. This representation of the cytokine modulation is shown with a promising approach in detail in the case of corneal transplantation by Reza Dana et al., using several animal models. They have shown the beneficial effects of low-dose IL-2 and IL-6 blocking antibody in their previous papers (69, 70). Secondly, after understanding of the involvement of the cytokines in several conditions of the diseases, one can predict/design a drug, using one cytokine or combination of the cytokines. This will certainly be a much safer option and will not just follow the mainstay of the treatment which in many cases is not safe and effective. One can take an example from anti-TNF treatment where 40% of patients have no response to the treatment and it is associated with some adverse effects like increased risk of infection, triggering of development of autoimmune diseases due to the global inhibition of TNF biological functions (71).

This study is a conglomeration of the observations from the publications of the last 20 years and gives a hint to the research direction in the field of ocular surface disorders. The number of references we have worked on did not represent a huge number (52) – this can be one of the limitations of the study. But, the number of animal models we have is relatively high (14) along with the diversity in different species following our search criteria. Another important point to note is that we only considered those studies for the therapeutic approach where the drugs are tested i*n vivo* for identification of immunological parameters. In this case, we have not included those *in vivo* studies where the animal models are only used for the tolerability assay analysis like the testing of Xiidra in pigs (72). Despite these two limitations, this study gives an indication which immune cells or which immune mediators are able to alleviate the ocular surface diseases. There are already existing conditional knockdown animal models (35, 41) with the deletion of genes for the important cytokine and chemokine factors like IL-1β and IFN-γ discussed in the manuscript and combinational treatment in these animal models along with dry eye conditions will give new insight to the field of research. The preponderance of the adaptive immune system factors in animal models is different than what we have observed from the meta-analysis of the human patients where we have found the predominance of dendritic cells (innate immune system) (7). It is true though that the dendritic cells are considered to be the part of the innate immune system, this is also an important cellular component to drive the adaptive T cells responses through their presentation of the antigens. What we are mentioning here is that our meta-analysis result is different between the human studies and animal models. This is a word of caution that no animal models are an exact representation of human diseases and depicts the challenge of the researchers involved in representing the multi-factorial human diseases.

## Summary

• DED animal models mostly show the predominance of Th1-modulatory and pro-inflammatory cytokines (IL-1β and TNF-α) despite the modulation either genetically or environmentally.
• It is clear at least from the current study that the blocking of pro-inflammatory cytokine (IL-1β and TNF-α) and induction of anti-inflammatory cytokine (IFN-γ, IL-12, and IL-4) might help to ameliorate the disease. An understanding of the delicate balance between Th1 and Th2 cells and their relationship with the pro- and anti-inflammatory cytokines will enrich the DED research.
• In the animal model induced by environmental factors, the innate immune pathway may play a more dominant effect than the adaptive immune system pathway.
• IL-17 though has shown to be important, but the distribution of this factor is not homogeneous among all the research studies. It can be an over-powering effect from a few selected papers. But an interesting point to note is that an understanding of the co-operative mechanism between the cytokines especially IL-6, TGF-b, and Th17 will foster DED therapy research.
• This study gives an insight into the treatment paradigm for chronic and acute DEDs in terms of the identification of immune factors in the autoimmune and environmental factor-induced animal models. This study also tries to identify the gap of knowledge in the specific therapeutic options for the DED.

## Data Availability Statement

All datasets generated for this study are included in the article/Supplementary Material.

## Author Contributions

TB designed the study and wrote the manuscript. CL did all the analysis and figure preparation. SD critically commented and helped in revising the manuscript. All authors contributed to the article and approved the submitted version.

## Conflict of Interest

The authors declare that the research was conducted in the absence of any commercial or financial relationships that could be construed as a potential conflict of interest.

## Footnotes

1 https://github.com/yollct/animal_studies_dry_eye.git
