# Supplementary material for: Cluster Analysis of Dry Eye Disease Models Based on Immune Cell Parameters – New Insight Into Therapeutic Perspective
Source: Front Immunol. 2020 Sep 29;11:1930. doi: 10.3389/fimmu.2020.01930 (PMC7550429; doi:10.3389/fimmu.2020.01930)
Supplement: Supplementary file 1 [file Data_Sheet_1.pdf]

Supplementary materials

Study	Dry eye model Total	Dry eye model Mean	Dry eye model SD	Normal control Total	Normal control Mean	Normal control SD	SMD	95%-CI	Weight
chauhan 2009_IL-17_C57BL/6 mice	5	2.50	0.50	5	1.00	0.10	3.76	[1.31; 6.21]	1.4%
chauhan 2009_IL-6_C57BL/6 mice	5	1.30	0.05	5	1.00	0.10	3.43	[1.13; 5.72]	1.5%
chen 2013_TNF-α_C57BL/6 mice	3	9.00	1.00	3	1.00	0.10	9.01	[0.16; 17.85]	0.4%
chen 2013_IL-17_C57BL/6 mice	3	28.00	5.00	3	1.00	0.10	6.11	[0.00; 12.22]	0.6%
chen 2013_IL-6_C57BL/6 mice	3	11.00	1.00	3	1.00	0.10	11.26	[0.27; 22.25]	0.3%
chen 2013_IL-1β_C57BL/6 mice	3	8.70	2.00	3	1.00	0.10	4.35	[-0.14; 8.85]	0.9%
chen 2014_TNF-α_C57BL/6 mice	3	9717.00	704.32	3	1066.00	106.59	13.74	[0.38; 27.10]	0.2%
chen 2014_IL-1β_C57BL/6 mice	3	757.73	23.74	3	353.60	26.36	12.89	[0.34; 25.44]	0.2%
De Paiva 2006_IL-1α_C57BL/6 mice	5	1.60	0.10	5	1.00	0.10	5.42	[2.13; 8.71]	1.2%
De Paiva 2006_IL-1β_C57BL/6 mice	5	1.70	0.40	5	1.00	0.10	2.17	[0.43; 3.91]	1.6%
el annan 2009_IFN-γ_C57BL/6 mice	4	210.00	20.00	4	90.00	10.00	6.60	[1.85; 11.35]	0.8%
el annan 2009_IL-4_C57BL/6 mice	4	30.00	5.00	4	55.00	5.00	-4.35	[-7.64; -1.05]	1.2%
ji 2013_IFN-γ_C57BL/6 mice	3	10.00	3.00	3	1.00	0.10	3.39	[-0.25; 7.04]	1.1%
ji 2013_IL-6_C57BL/6 mice	3	2.50	0.30	3	0.20	0.00	8.67	[0.15; 17.20]	0.4%
ji 2013_IL-21_C57BL/6 mice	3	25.00	2.00	3	3.00	0.00	12.45	[0.32; 24.57]	0.2%
kodati 2014_CCR1_C57BL/6 mice	6	1800.00	500.00	6	250.00	10.00	4.05	[1.77; 6.32]	1.5%
kodati 2014_CCR2_C57BL/6 mice	6	2700.00	500.00	6	1200.00	100.00	3.84	[1.65; 6.03]	1.5%
kodati 2014_CCR5_C57BL/6 mice	6	3700.00	1000.00	6	1600.00	200.00	2.69	[0.96; 4.42]	1.6%
kodati 2014_CCR7_C57BL/6 mice	6	2500.00	500.00	6	250.00	10.00	5.87	[2.79; 8.96]	1.2%
Lee 2017_dendritic cells_C57BL/6 mice	4	160.00	50.00	4	90.00	10.00	1.69	[-0.12; 3.50]	1.6%
Lee 2017_dendritic cells_C57BL/6 mice	4	160.00	50.00	4	80.00	5.00	1.96	[0.03; 3.89]	1.5%
lee 2012_TLR4_C57BL/6 mice	6	28.00	2.00	6	5.00	3.00	8.33	[4.11; 12.55]	1.0%
li 2019_IL-1β_C57BL/6 mice	6	115.00	20.00	6	40.00	0.00	4.90	[2.25; 7.54]	1.4%
li 2019_IL-6_C57BL/6 mice	6	40.00	5.00	6	8.00	0.00	8.35	[4.12; 12.59]	1.0%
li 2019_IFN-γ_C57BL/6 mice	6	55.00	6.00	6	20.00	0.00	7.61	[3.73; 11.50]	1.0%
li 2019_TNF-α_C57BL/6 mice	6	20.00	5.00	6	5.00	0.00	3.92	[1.69; 6.14]	1.5%
li 2019_CD11b _C57BL/6 mice	6	800.00	50.00	6	100.00	1.00	18.27	[9.28; 27.26]	0.4%
sung 2015_CD4 T cells_C57BL/6 mice	5	64.36	15.19	5	16.05	5.22	3.84	[1.35; 6.33]	1.4%
sung 2015_IL-1β_C57BL/6 mice	5	118.00	5.00	5	60.00	5.00	10.48	[4.45; 16.50]	0.6%
sung 2015_IL-6_C57BL/6 mice	5	33.00	6.00	5	12.00	3.00	4.00	[1.43; 6.57]	1.4%
sung 2015_TNF-α_C57BL/6 mice	5	12.00	1.00	5	2.00	0.50	11.43	[4.87; 17.98]	0.6%
sung 2015_IFN-γ_C57BL/6 mice	5	60.00	6.00	5	36.00	5.00	3.93	[1.39; 6.46]	1.4%
yoon 2007_CXCR3_C57BL/6 mice	10	2.30	0.50	10	1.00	0.10	3.45	[1.97; 4.93]	1.7%
krauss 2015_dendritic cells_C57BL/6 mice	16	3.90	0.50	16	2.10	0.50	3.51	[2.36; 4.66]	1.7%
krauss 2015_dendritic cells_C57BL/6 mice	16	2.10	0.50	16	1.90	0.50	0.39	[-0.31; 1.09]	1.8%
wu 2017_CD4 T cells_C57BL/6 mice	5	32.00	5.00	5	9.50	0.10	5.75	[2.28; 9.21]	1.1%
wu 2017_IL-13_C57BL/6 mice	5	150.00	50.00	5	280.00	50.00	-2.35	[-4.16; -0.54]	1.6%
wu 2017_IFN-γ_C57BL/6 mice	5	1400.00	250.00	5	1000.00	100.00	1.90	[0.26; 3.53]	1.6%
wu 2017_IL-17A_C57BL/6 mice	5	900.00	80.00	5	600.00	50.00	4.06	[1.46; 6.66]	1.4%
choi 2016_CD4 T cells_C57BL/6 mice	10	65.76	10.47	10	22.54	3.74	5.27	[3.24; 7.29]	1.5%
choi 2016_IL-1β_C57BL/6 mice	10	100.00	20.00	10	50.00	5.00	3.29	[1.85; 4.72]	1.7%
choi 2016_IL-6_C57BL/6 mice	10	30.00	2.00	10	19.00	3.00	4.13	[2.46; 5.81]	1.6%
choi 2016_TNF-α_C57BL/6 mice	10	15.00	3.00	10	3.50	0.10	5.19	[3.19; 7.19]	1.5%
choi 2016_IFN-γ_C57BL/6 mice	10	78.00	10.00	10	21.00	2.00	7.57	[4.81; 10.33]	1.3%
Dong 2010_TNF-α_C57BL/6 mice	3	0.80	0.40	3	0.20	0.10	1.65	[-0.61; 3.90]	1.5%
Dong 2010_CCL2_C57BL/6 mice	3	0.60	0.20	3	0.20	0.01	2.26	[-0.45; 4.97]	1.3%
Dong 2010_CXCL2_C57BL/6 mice	3	0.60	0.20	3	0.05	0.03	3.08	[-0.30; 6.45]	1.2%
Dong 2010_KC_C57BL/6 mice	3	1.20	0.30	3	0.10	0.05	4.09	[-0.17; 8.35]	0.9%
Dong 2010_INOS_C57BL/6 mice	3	0.50	0.50	3	0.01	0.00	1.11	[-0.82; 3.03]	1.6%
Reins 2017_IL-1α_C57BL/6 mice	5	29.00	2.00	5	26.00	5.00	0.71	[-0.59; 2.01]	1.7%
Reins 2017_IL-2_C57BL/6 mice	5	8.00	3.00	5	6.00	2.00	0.71	[-0.59; 2.01]	1.7%
Reins 2017_CXCL1_C57BL/6 mice	5	20.00	5.00	5	10.00	4.00	1.99	[0.32; 3.67]	1.6%
Reins 2017_IL-9_C57BL/6 mice	5	200.00	10.00	5	150.00	50.00	1.25	[-0.17; 2.68]	1.7%
De Paiva 2008_IL-6_C57BL/6 mice	8	2.51	0.57	8	1.00	0.36	2.99	[1.45; 4.54]	1.6%
De Paiva 2008_TGF-β_C57BL/6 mice	8	0.47	0.32	8	1.00	0.29	-1.64	[-2.82; -0.46]	1.7%
De Paiva 2008_IL-23_C57BL/6 mice	8	2.24	0.51	8	1.00	0.49	2.34	[0.99; 3.70]	1.7%
De Paiva 2008_IL-17A_C57BL/6 mice	8	1.89	0.66	8	1.00	0.55	1.39	[0.26; 2.51]	1.7%
De Paiva 2008_IL-15_C57BL/6 mice	8	0.44	0.27	8	1.00	0.21	-2.19	[-3.50; -0.88]	1.7%
De Paiva 2008_CCL20_C57BL/6 mice	8	2.52	0.44	8	1.00	0.52	2.98	[1.44; 4.53]	1.6%
De Paiva 2008_IFN-γ_C57BL/6 mice	8	0.21	0.54	8	1.00	0.35	-1.64	[-2.82; -0.46]	1.7%
De Paiva 2008_IL-12_C57BL/6 mice	8	0.37	0.31	8	1.00	0.51	-1.41	[-2.54; -0.28]	1.7%
De Paiva 2008_IL-2_C57BL/6 mice	8	1.29	0.35	8	1.00	0.67	0.51	[-0.49; 1.51]	1.8%
Qu 2019_IL-1β_C57BL/6 mice	5	2.50	1.00	5	1.00	0.10	1.91	[0.27; 3.55]	1.6%
Qu 2019_IL-6_C57BL/6 mice	5	7.00	3.00	5	1.00	0.10	2.55	[0.66; 4.45]	1.6%
Qu 2019_IL-17A_C57BL/6 mice	5	8.00	2.00	5	1.00	0.10	4.47	[1.66; 7.27]	1.3%
Qu 2019_IFN-γ_C57BL/6 mice	5	2.80	0.50	5	1.00	0.10	4.51	[1.68; 7.33]	1.3%
Chen and chauhan 2017_TH17 cells_C57BL/6 mice	5	3.90	0.50	5	0.30	0.10	9.02	[3.79; 14.24]	0.8%
Chen and chauhan 2017_IL-17_C57BL/6 mice	5	40.00	10.00	5	1.00	0.10	4.98	[1.92; 8.05]	1.2%
Chen and chauhan 2017_IL-15_C57BL/6 mice	5	100.00	20.00	5	45.00	5.00	3.41	[1.12; 5.69]	1.5%
Zhang 2011_CD4 T cells_C57BL/6 mice	3	25.00	2.00	3	14.00	2.00	4.40	[-0.14; 8.94]	0.9%
Zhang 2011_IFN-γ_C57BL/6 mice	3	19.00	1.00	3	5.00	1.00	11.20	[0.27; 22.13]	0.3%
Zhang 2011_IL-17A_C57BL/6 mice	3	28.00	1.00	3	16.00	1.00	9.60	[0.19; 19.01]	0.3%
Zhang 2011_IL-13_C57BL/6 mice	3	20.00	2.00	3	38.00	1.00	-9.11	[-18.05; -0.17]	0.4%
Coursey 2013_CD4 T cells_C57BL/6 mice	3	2.00	0.10	3	1.20	0.20	4.05	[-0.18; 8.27]	1.0%
Coursey 2013_IFN-γ_C57BL/6 mice	3	2.30	0.50	3	1.00	0.10	2.88	[-0.33; 6.10]	1.2%
Coursey 2013_IL-17A_C57BL/6 mice	3	1.80	0.30	3	1.00	0.10	2.86	[-0.33; 6.06]	1.2%
Coursey 2013_IL-13_C57BL/6 mice	3	1.90	0.20	3	1.00	0.10	4.55	[-0.13; 9.23]	0.9%
McClellan 2014_CD4 T cells_C57BL/6 mice	3	1.50	0.30	5	0.50	0.10	4.54	[1.11; 7.98]	1.1%
McClellan 2014_CD8 T cells_C57BL/6 mice	3	0.14	0.30	5	0.80	0.10	-3.00	[-5.51; -0.49]	1.4%
McClellan 2014_IL-17A_C57BL/6 mice	3	13.00	1.00	3	1.00	0.10	13.51	[0.37; 26.65]	0.2%
McClellan 2014_CCL20_C57BL/6 mice	3	4.00	1.00	3	1.00	0.10	3.38	[-0.26; 7.01]	1.1%
McClellan 2014_IFN-γ_C57BL/6 mice	3	3.00	1.50	3	1.00	0.10	1.51	[-0.66; 3.67]	1.5%
**Random effects model**	**441**			**445**			**3.08**	**[2.48; 3.67]**	**100.0%**

Heterogeneity: $I^2 = 82\%$, $\tau^2 = 4.9514$, $p < 0.01$

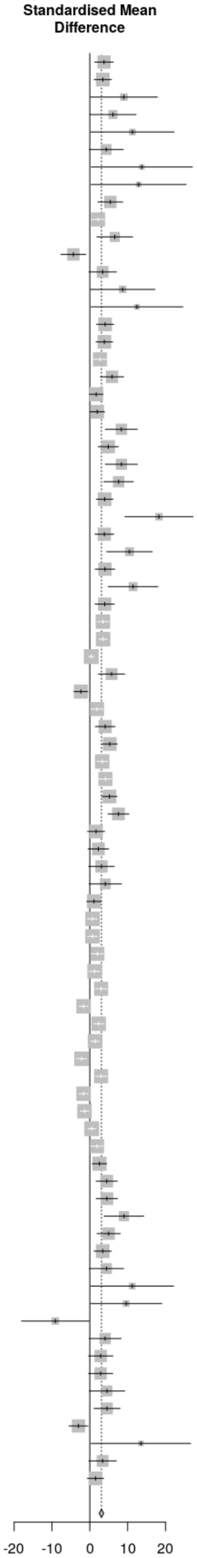

Supplementary Figure 1. Meta-analysis for C57BL/6 mice.

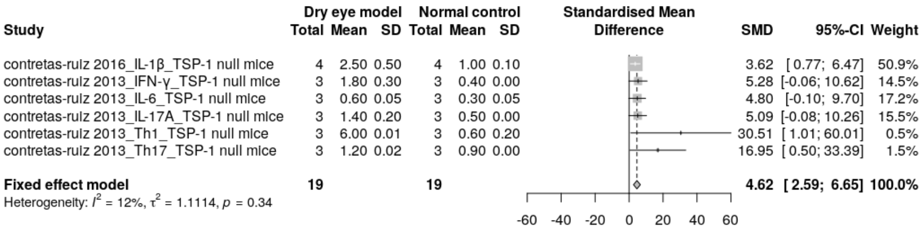

Supplementary Figure 2. Meta-analysis for TSP-1 null mice.

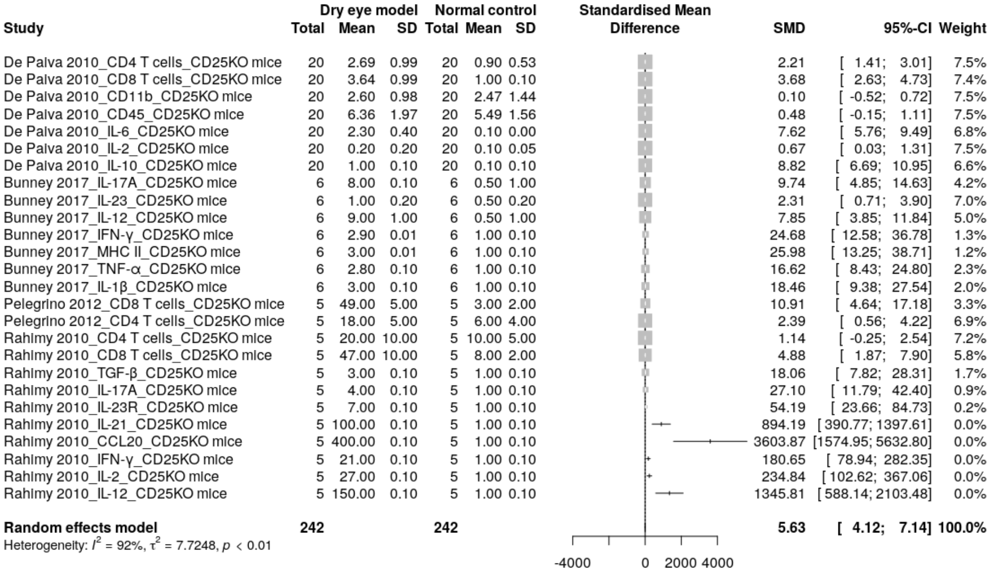

Supplementary Figure 3. Meta-analysis for CD25KO mice.

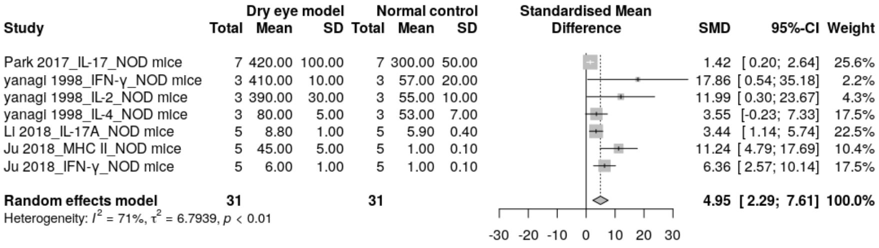

Supplementary Figure 4. Meta-analysis for NOD mice.

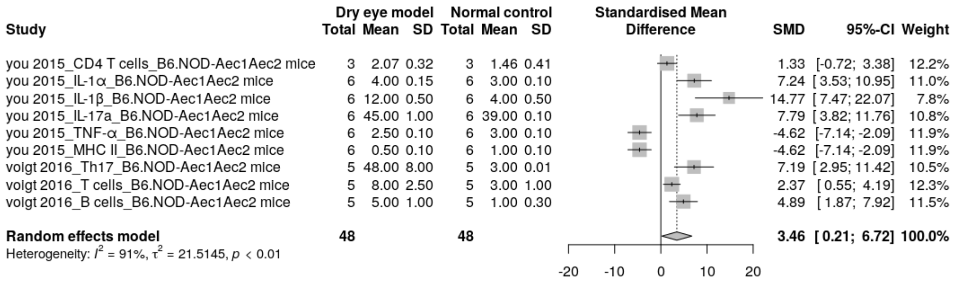

Supplementary Figure 5. Meta-analysis for B6.NOD-Aec1Aec2 mice.

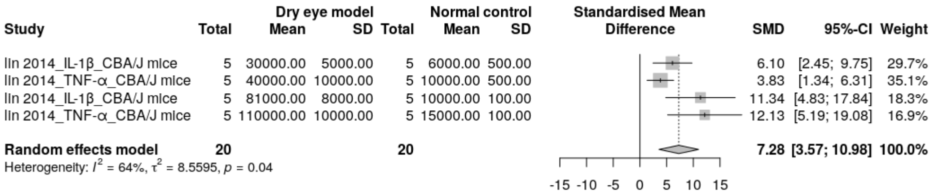

Supplementary Figure 6. Meta-analysis for CBA/J mice.

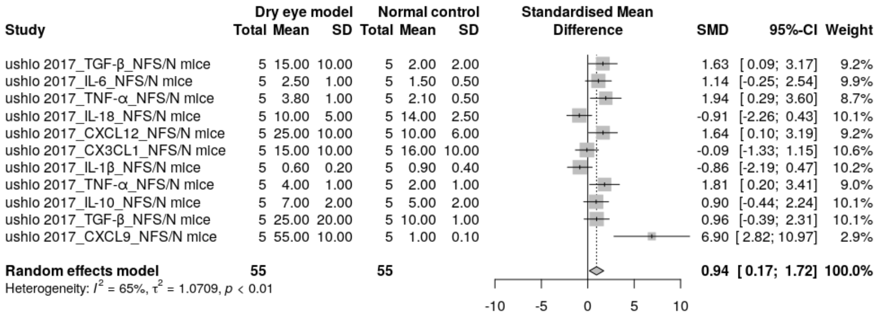

Supplementary Figure 7. Meta-analysis for NFS/N mice.

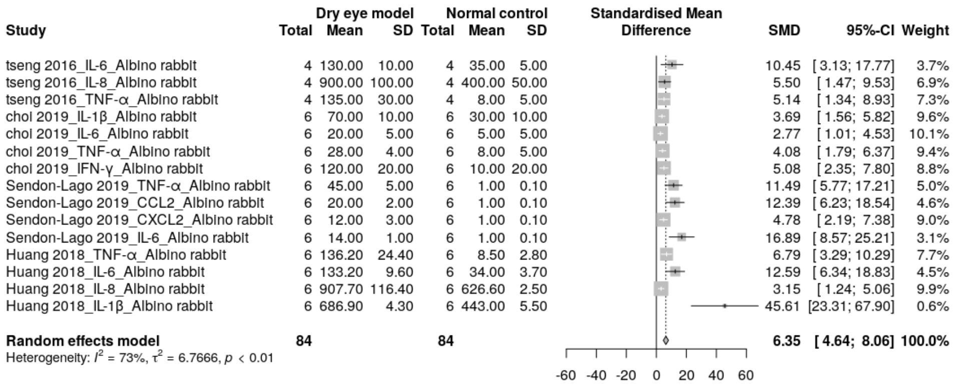

Supplementary Figure 8. Meta-analysis for Albino rabbit.

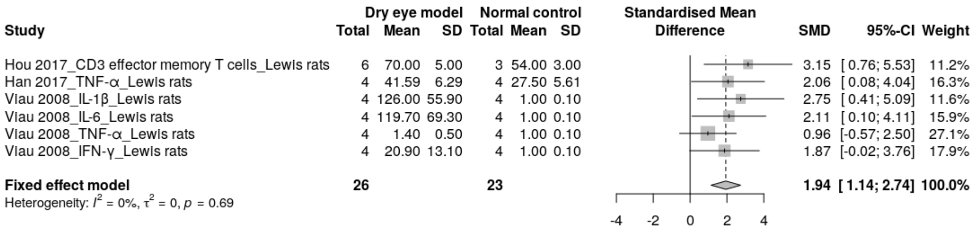

Supplementary Figure 9. Meta-analysis for Lewis rats.

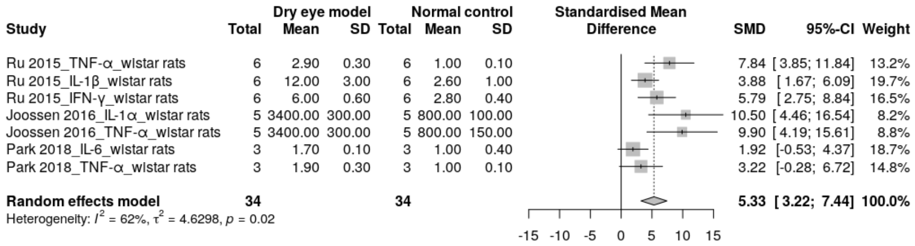

Supplementary Figure 10. Meta-analysis for Wistar rats.

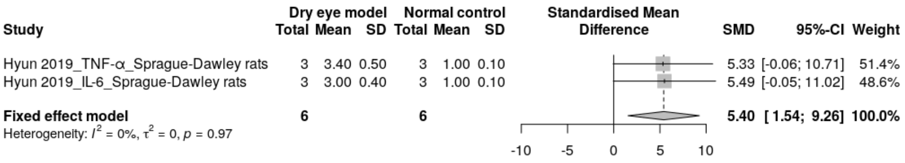

Supplementary Figure 11. Meta-analysis for Sprague-Dawley rats.

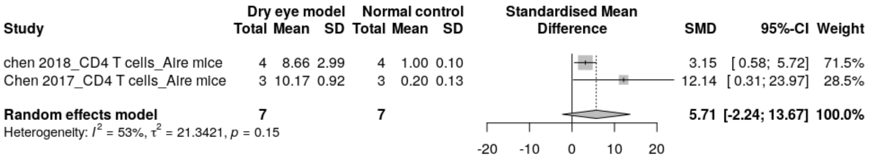

Supplementary Figure 12. Meta-analysis for Aire mice.

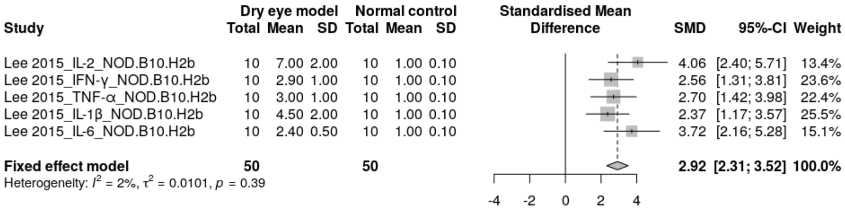

Supplementary Figure 13. Meta-analysis for NOD.B10.H2b.

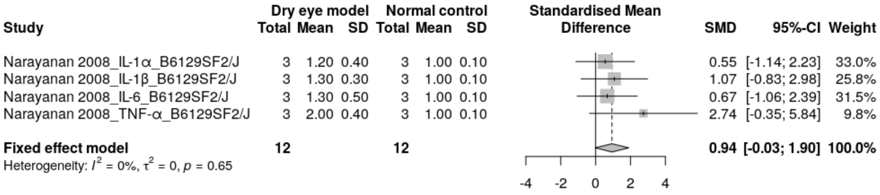

Supplementary Figure 14. Meta-analysis for B6129SF2/J.

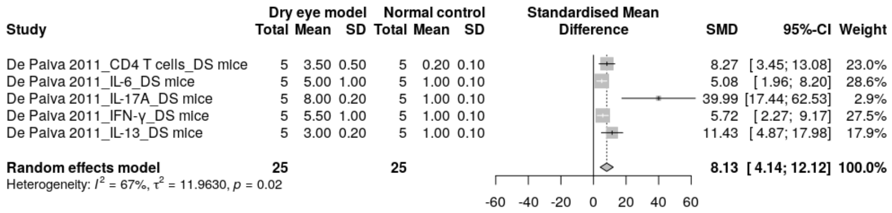

Supplementary Figure 15. Meta-analysis for DS mice.

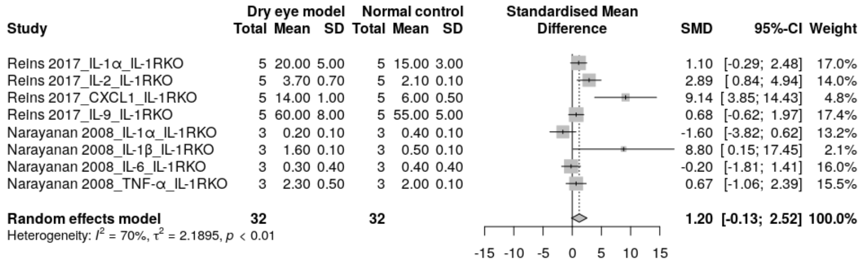

Supplementary Figure 16. Meta-analysis for IL-1RKO.

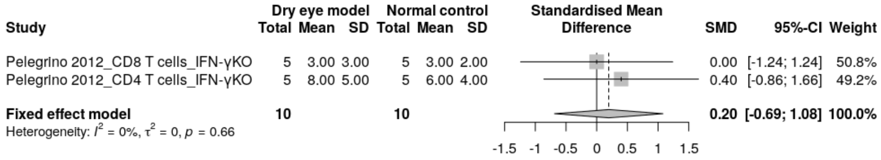

Supplementary Figure 17. Meta-analysis for IFN-γKO.
